# Kilonova simulations: connecting observations with the underlying physics

**DOI:** 10.1098/rsta.2024.0119

**Published:** 2025-05-01

**Authors:** Christine Collins, Luke Shingles, Vimal Vijayan

**Affiliations:** ^1^ Trinity College Dublin, Dublin, Ireland; ^2^ GSI Helmholtz Centre for Heavy Ion Research GmbH, Darmstadt, Hessen, Germany

**Keywords:** kilonova, radiative transfer, neutron star mergers, simulations

## Abstract

Kilonova observations contain information about heavy-element r-process nucleosynthesis and the behaviour of high-density matter. However, interpreting what these observations tell us about the underlying physics requires detailed modelling. We outline recent kilonova radiative transfer simulations that are based on hydrodynamical models of neutron star merger ejecta. The simulated spectra in the polar directions show a remarkably similar evolution to the observations of AT2017gfo. Using these simulations, we show the importance of accurate atomic data for kilonova modelling, as well as the importance of three-dimensional simulations. By improving radiative transfer simulations and by extending this study to consider a range of theoretical equations of state, simulations will be able to connect observations to the underlying merger physics and place constraints on the high-density equation of state and r-process nucleosynthesis.

This article is part of the Theo Murphy meeting issue ‘Multi-messenger gravitational lensing (Part 2)’.

## 1. Introduction

During the merging of binary neutron stars, the extreme, neutron-rich conditions allow the rapid neutron capture process (r-process) to take place [1–3]. The decay of the freshly synthesized radioactive r-process nuclei power a rapidly evolving kilonova transient [4–6]. By studying kilonovae, we have an opportunity to probe the origin of the heaviest elements in the Universe, and to understand r-process nucleosynthesis. The production of r-process elements is dependent on the equation of state of dense nuclear matter in the merging neutron stars. However, a broad range of theoretical equations of state is compatible with current observational constraints. Kilonova observations offer the potential to place constraints on the behaviour of high-density matter, for example, by connecting merger simulations using different equations of state to the observational signatures. To place constraints on r-process nucleosynthesis and on the behaviour of dense nuclear matter from kilonova observations, the observations must first be linked to the underlying merger physics through detailed simulations. In this article, we outline recent work in this direction and highlight the greatest uncertainties challenging current kilonova radiative transfer simulations, which need to be improved upon to place constraints on r-process nucleosynthesis and the equation of state from kilonova observations.

The kilonova AT2017gfo [7,8], associated with the gravitational wave signal GW170817 [9], was identified to be the electromagnetic counterpart of merging binary neutron stars. The extensive observations of AT2017gfo have provided an excellent dataset of kilonova light curves and spectra, the analysis and interpretation of which is still ongoing. The decline of the bolometric (ultraviolet–infrared) light curve of AT2017gfo is consistent with the decay of material that has undergone r-processing, providing confirmation that kilonovae are an astrophysical site for the production of r-process elements [7,10]. AT2017gfo was initially brightest in blue light curve bands and within days became brightest in the red bands [11]. The initial blue colours suggest the presence of light r-process material, with relatively low opacities allowing radiation to escape at blue optical wavelengths [8,10,12,13]. The long-lived red emission suggests the presence of heavy r-process elements, with much larger opacities that block radiation from escaping at short wavelengths. This absorbed radiation is then emitted at longer (redder) wavelengths through fluorescence.

Initial interpretations of AT2017gfo were made using simplified models, such as one-dimensional models with ‘blue’ or ‘red’ opacities, representative of light or heavy r-process material, respectively [8,10]. From these analyses, it was noted that a combination of these models could account for the observations of AT2017gfo, where the early light curves and spectra required light r-process material, while the later observations required heavier r-process material. This led to the interpretation that two distinct components were observed in AT2017gfo.

Detailed spectra were obtained for AT2017gfo, where the strongest spectral feature has been attributed to Sr II P-Cygni feature [14], supporting the evidence for light r-process material in the kilonova ejecta. Since this study, radiative transfer simulations have also found that the feature is consistent with Sr II [15,16]. However, there have also been suggestions that He I could explain this feature [17]. Additional features have had line identifications suggested, including La III, Ce III [18], Te III [19,20] and Y II [21]. Identifying spectral features and inferring abundances for AT2017gfo remain ongoing work. A number of possible kilonovae have also been identified following gamma-ray bursts [22–25] and provide further evidence for heavy-element nucleosynthesis in kilonovae.

To model kilonovae, assumptions must be made about the underlying merger ejecta, the r-process nucleosynthesis determining the ejecta composition, and the radiative transfer calculating the light curves and spectra produced for the kilonova model. One approach to kilonova modelling is a ‘backward modelling’ approach, where starting with the observations, the underlying composition required to reproduce the observations is inferred (e.g. [7,14]). Typically such backward modelling approaches assume simplifications in the density structure and r-process abundances, and the ejecta model is freely varied to match the observations. Kilonova spectra and light curves, however, are a complicated problem with many degeneracies and many parameters contributing to the spectrum formation. Another approach is a ‘forward modelling’ approach, typically starting with simulated merger ejecta and r-process compositions (e.g. [26–32]). Radiative transfer calculations are then carried out based on the ejecta composition and density structure. The advantage of this is that the number of assumptions made about the underlying ejecta are reduced. In this article we focus on a forward modelling approach.

## 2. Kilonova modelling

Using the examples of kilonova simulations carried out within our group, we now highlight uncertainties remaining in kilonova modelling and challenges in improving on these. The end goal of such kilonova simulations is to be able to constrain the high-density equation of state and r-process nucleosynthesis from kilonova observations.

### (a) Overview of kilonova simulations

**Figure 1.**
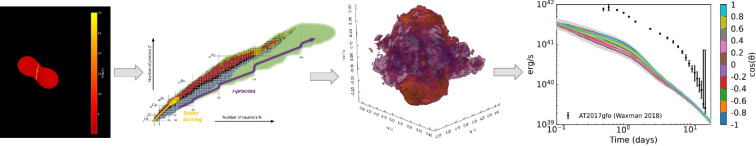
Self-consistent modelling pipeline for simulating kilonovae. A hydrodynamical neutron star merger simulation is carried out. Following this, r-process nuclear network calculations are carried out based on the merger simulation to calculate the nucleosynthetic abundances (image credit: EMMI, GSI/Different Arts). A snapshot of the merger simulation; the nucleosynthetic abundances are used as input to the radiative transfer calculation (shown is the density structure of the ejecta snapshot). The radiative transfer simulation produces light curves and spectra that can be directly compared to kilonova observations. Shown are simulated light curves compared to AT2017gfo (figure adapted from [31]).

The simulations we discuss in this article (originally presented by [31–33]) use a forward modelling approach to self-consistently simulate kilonovae from the merger through to the observations (figure 1). The ejecta used as input to the radiative transfer simulations comes from hydrodynamical merger simulations. Nucleosynthesis postprocessing calculations are carried out to calculate the r-process abundances and energy released from radioactive decays. A snapshot of the merger ejecta as well as the nucleosynthetic abundances are mapped on to the radiative transfer grid (see [31,32] for details). Radiative transfer simulations are then carried out, with atomic data used as input. For kilonova r-process-rich ejecta, this includes millions of energy levels.

The time-dependent, multi-dimensional radiative transfer code artis [32,34–36] is used to carry out the kilonova simulations discussed in this article. The radiative transfer simulations assume local thermodynamic equilibrium (LTE) and use a line-by-line Sobolev opacity treatment. The line-by-line treatment allows spectral features to be directly associated with the species responsible for their formation and importantly allows a treatment of fluorescence to be included in the simulation. Our atomic dataset is based on the Japan–Lithuania database [37], which covers ionization stages from neutral to triply ionized. We note that at very early times the ejecta are probably more highly ionized than this [13] and therefore our initial opacities may be underestimated.

From 0.1 days after the merger, radioactive decays are followed in the radiative transfer simulation, providing time-dependent thermalization of decay products and a time-dependent composition [32], since the radioactive decays occur on kilonova timescales.

The merger simulation considered here (described by [31]) is of equal mass 1.35 M_ʘ_ neutron stars using the SFHo [38] equation of state. It was carried out with a three-dimensional general relativistic smoothed-particle hydrodynamics (SPH) code [39–41] and included an advanced neutrino leakage treatment (improved leakage-equilibration-absorption scheme (ILEAS)) [42]. The hydrodynamical simulation followed the evolution until 20 ms after the merger, and therefore only includes dynamical ejecta. We note that the ejecta are not yet homologously expanding. However, an assumption of the radiative transfer simulation is that the ejecta are in homologous expansion. As described by Collins *et al*. [31], we allow the SPH particles to propagate ballistically for 0.5 s after the end of the SPH simulation to allow the ejecta to expand according to the final velocity of each particle, and from this point we assume homologous expansion. The total mass of ejecta from this simulation is 0.005 M_ʘ_. It is expected that matter ejection will continue after this simulation is stopped; however, long-term evolution simulations are required to follow the hydrodynamics beyond this time (e.g. see [43,44]). In the future, it will be important to investigate the effect of later ejecta components on the kilonova signature, for example using models such as the end-to-end models of Just *et al*. [43]. Since our model includes only dynamical ejecta, it is likely that the total mass, and therefore brightness, would be higher if secular ejecta were included. We note, however, that approximate calculations including a secular ejecta component for this merger model indicate that the early kilonova may be dominated by the dynamical ejecta component [31].

### (b) Importance of three-dimensional simulations

Kilonova ejecta from merger simulations show a high degree of asymmetry, both in the density structure and in the nucleosynthetic abundances. In the ejecta model we consider (described by [31–33]), the densities are typically lower near the poles compared to at the equator in the plane of rotation, and the electron fraction ($Y_{e}$) is higher near the poles (see fig. 1 of [31]). As a result, lighter r-process material is synthesized near the pole, while lanthanide-rich material is present near the equator where the ejecta have a lower $Y_{e}$. The combination of density and $Y_{e}$ leads to lower optical depths in the directions of the poles, which are therefore brighter than viewing directions near the equator [31,32]. It was inferred that AT2017gfo shows a high degree of sphericity [45] when comparing independent measurements of the photospheric velocity most sensitive to the expansion towards an observer and the expansion perpendicular to an observer. Despite the asymmetry of the merger ejecta, our kilonova simulation also demonstrated a similarly high degree of sphericity when the same measurements were made for the synthetic spectra [33]. The level of asymmetry shown by the ejecta model is therefore compatible with the constraints inferred for AT2017gfo.

**Figure 2.**
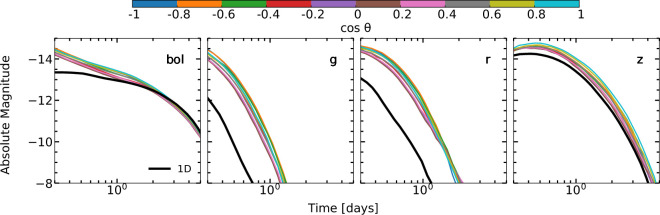
Kilonova light curves for a three-dimensional simulation compared to a one-dimensional simulation where the same ejecta have been spherically averaged. The one-dimensional simulation is unable to reproduce the light curves of any direction in the three-dimensional model. Shown are the bolometric and *grz*-band light curves for models three-dimensional AD2 and one-dimensional AD2 [32]. The colour bar indicates the viewing angle in the three-dimensional simulation while the black lines show the one-dimensional simulation.

Shingles *et al*. [32] demonstrated the importance of simulating kilonova radiative transfer in three dimensions. They show that a one-dimensional simulation of spherically averaged merger ejecta produces light curves and spectra that do not resemble any line of sight in the three-dimensional simulation. The light curves for the three-dimensional model compared to the one-dimensional model are shown in figure 2 (see also fig. 5 of [32] for spectra). The one-dimensional model is unable to reproduce any line of sight in the three-dimensional model. In bolometric light, the one-dimensional model is initially fainter than the three-dimensional model. Once it becomes optically thin, the bolometric light curve converges to the rate of thermalized energy, similar to the three-dimensional simulation. The one-dimensional model is substantially redder than the three-dimensional model, showing light curves much fainter than the three-dimensional model in *g* and *r* bands. It is important to consider that the difference in brightness between the one- and three-dimensional models would imply different ejecta masses and would underestimate the amount of light r-process material present in the ejecta while potentially overestimating the amount of lanthanide-rich material.

**Figure 3.**
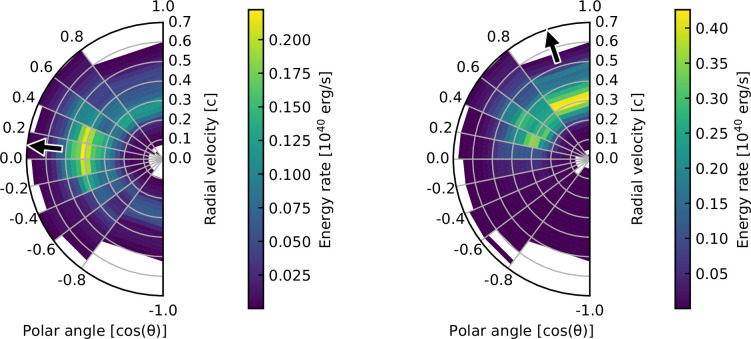
Ejecta locations where radiation last interacted before escaping from the ejecta, arriving at an observer at 0.4 days after the merger. This gives an indication of the ejecta regions responsible for forming a spectrum viewed by an observer in the directions indicated by the arrows. The radial velocity and polar angle of the last interaction location are plotted, showing radiation that escaped towards an observer in the direction of the equator (left) or in the direction of the northern pole (right). The shaded regions show the extremely broad range of ejecta from which radiation escapes towards an observer. For example, an observer viewing towards the equator will see not only radiation coming from equatorial ejecta but also radiation from ejecta near the poles. Figure taken from [33].

Low $Y_{e}$, lanthanide-rich material is present near the equator in the ejecta model (see [31]), and yet the three-dimensional model shows much bluer colours than the one-dimensional model, even in directions where red ejecta are present. In the radiative transfer simulation, radiation is able to escape from an extremely broad range of ejecta (see [33]). As can be seen in figure 3, an observer viewing towards the equator will see radiation that has escaped from equatorial ejecta, but will also see radiation that has escaped from ejecta in regions near the poles. This leads to brighter and bluer light curves than if only lanthanide-rich material were present in the ejecta.

### (c) Importance of accurate atomic data

**Figure 4.**
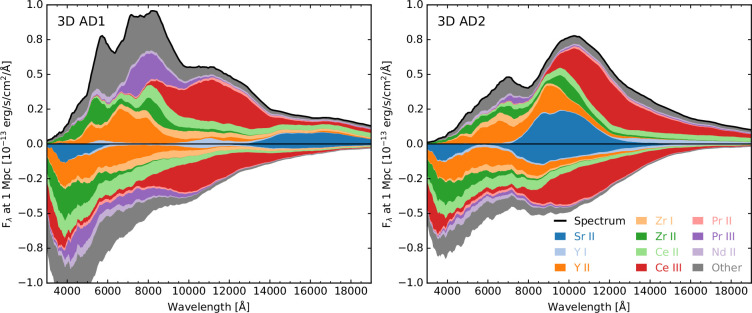
Spectra at 0.8 days in the polar direction (−1≤ cos θ<−0.8) where only the atomic data included in the radiative transfer simulation has changed [32]. The spectra in the left panel were produced using the AD1 atomic dataset, which comprised theoretically calculated atomic data [37] that have not been calibrated to experimental values. The right panel shows the effect of replacing the atomic data in AD1 for Sr, Y and Zr with experimental values sourced from Kurucz [47]. This figure has been adapted from fig. 3 of [32].

To calculate kilonova spectra and light curves, we must know the atomic data for the elemental species synthesized in the kilonova ejecta. However, the known atomic data for r-process elements are highly incomplete. For many elements, no experimental data are available, and where experimental data do exist, many energy levels are missing. Because of this, theoretical atomic datasets have been calculated to provide the missing atomic data (e.g. [37]); however, there are uncertainties in these calculations that lead to energy level values that are shifted from the real values. The result of this in radiative transfer calculations is that spectral features form at incorrect wavelengths. The effect of inaccurate atomic data on the spectra has been demonstrated [32] by comparing a simulation using only theoretical atomic data (AD1) from the Japan–Lithuania Opacity Database [37,46] to a simulation using atomic data that are the same as AD1, except that the data for Sr, Y and Zr are replaced with data from the Kurucz [47] extended line list, which includes calibrated wavelengths where available. Figure 4 shows the effect on the spectra of changing only the atomic data of Sr, Y and Zr.

The strongest feature in AT2017gfo has been identified as the Sr II triplet with wavelengths of 10036.65, 10327.31 and 10914.87. In the uncalibrated AD1 dataset, this feature forms around 15 000−20 000. In the simulation with the AD2 dataset, this feature forms at the correct (rest) wavelengths and shows good agreement with the feature observed for AT2017gfo. We note that the relative contributions of ions for which we kept the atomic data the same also change when using the AD2 instead of AD1 atomic dataset. For example, Pr III and Ce III (see figure 4). Since individual spectral features are not isolated, this demonstrates that having correct atomic data for all species will probably be important.

Changing the atomic data not only affects the formation of specific spectral features but also changes the overall spectral energy distribution (SED). With the uncalibrated atomic data (AD1), the spectrum peaks at bluer wavelengths. This highlights that accurate atomic data are important not only for identifying spectral features but also for calculating the SED and colour temperature evolution. The blue emission has been used to infer the amount of light r-process material in AT2017gfo. These simulations show that the blue emission is sensitive to the accuracy of the atomic data. To make accurate measurements of r-process abundances, we first need accurate atomic data.

In addition to accurate atomic data, we also require complete atomic data. The forest of lines of r-process elements provide opacity, and without a complete atomic line list, the overall opacity will be incorrect. Currently, theoretical datasets are somewhat complete; however, they are insufficiently accurate. Experimentally obtained data provide the accuracy required; however, experimental data are very incomplete and not able to provide the overall opacity in many cases. For calculating kilonova spectra, accurate and complete atomic data are critical.

These radiative transfer simulations have been carried out assuming LTE. This is probably a reasonable assumption at early times while the ejecta are dense. However, due to the extremely high expansion velocities, kilonovae will quickly transition to a regime where non-LTE processes become important (a few days to weeks after the merger) [48]. By assuming LTE, the Boltzmann and Saha equations can be used to calculate the excitation and ionization state of the ejecta. Using this assumption, only energy levels and A-values are required for radiative transfer calculations. However, in non-LTE conditions, the ionization and excitation state must be calculated from the radiative and collisional rates. This requires significantly more atomic data (e.g. collisional and photoionization cross section), which are even more sparsely known for r-process elements than the energy levels. The assumption of LTE may introduce uncertainties in our radiative transfer simulations; however, to investigate the importance of non-LTE will require significantly more atomic data than is currently known.

### (d) Comparison to AT2017gfo

**Figure 5.**
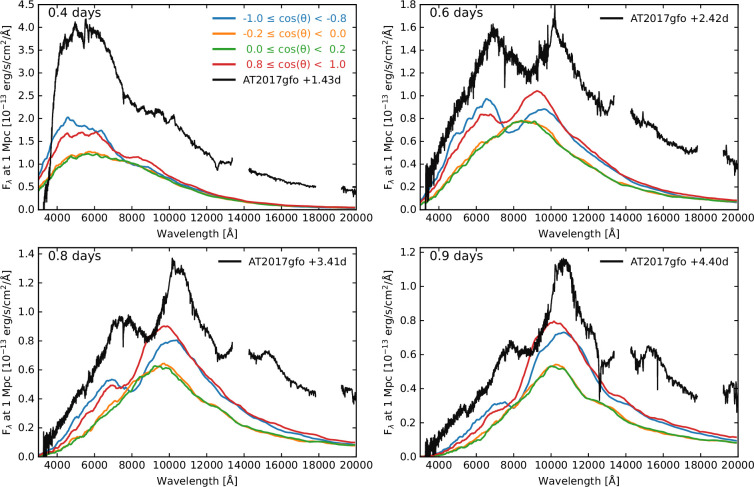
Simulated spectra (model three-dimensional AD2) in the directions of the poles and near the equator, compared to observations of AT2017gfo. The simulated spectra show a similar evolution to AT2017gfo in the polar directions, although at earlier times. The times of the simulated spectra after the merger are indicated in each panel, as well as the observed times of the spectra for AT2017gfo after the merger. The spectra at the equator are relatively featureless. Figure partially from [33].

We note that since our ejecta model includes only dynamical ejecta, and that the mass is approximately 10 times lower than that inferred for AT2017gfo, we do not expect a direct match between our simulation and the observations. Nonetheless, in this section, we discuss the similarities between the simulated and observed spectra. The three-dimensional simulation using the AD2 atomic data (three-dimensional AD2 [32]) is compared to the spectra observed for AT2017gfo in figure 5. The simulation shows a similar spectral evolution in the polar directions to that observed for AT2017gfo. Both AT2017gfo and the simulated spectra near the poles are initially relatively featureless and show an Sr II feature that grows in strength with time (see [32] and [33] for details). However, the simulated spectra evolve much faster than AT2017gfo. The simulated spectra at 0.4 days after the merger in the polar directions are comparable to the spectrum observed at 1.43 days for AT2017gfo, while the simulated 0.6 day spectra are comparable to the observed spectrum at 2.42 days. Given the lower total ejecta mass and that the model includes only dynamical ejecta, the faster evolution of the simulated spectra is probably not surprising. However, future simulations of models with higher ejecta masses and all ejecta components are required to investigate this further. As a result of the lower ejecta mass, our kilonova simulation is fainter than AT2017gfo, since the model has lower masses of radioactive material in the ejecta. The bolometric luminosity is proportional to the ejected mass of radioactive r-process material. Considering that our model was not chosen to try to match AT2017gfo, nor tuned in any way, we find remarkably good agreement in the spectral evolution.

While the spectra at the poles resemble the observations of AT2017gfo, the spectra predicted near the equator are relatively featureless. The emission and absorption features are extremely broad due to the high expansion velocities, and at the equator, the overlapping features blend together. This gives a prediction that a kilonova viewed from near the equator will appear different to AT2017gfo and may be relatively featureless [32]. It also suggests that colour temperature may not necessarily be significantly redder at early times than AT2017gfo, despite low $Y_{e}$, lanthanide-rich material being present near the equator.

While we have only studied one model at this time, more models could help predict the variation we can expect in kilonova observations. Such predictions could help with the identification of electromagnetic counterparts from gravitational wave signals. Particularly in the context of lensed kilonovae, since these will probably be at high redshift and the observations would then be most sensitive to the blue end of the kilonova spectrum. Kilonova simulations will be able to predict whether a blue component is expected in all kilonovae, as well as the timescales for which a blue component is observable. This simulation suggests that a blue component would be observable in all viewing directions, even where lanthanide-rich material is present. However, more models are required to determine whether this is a general prediction for all kilonovae.

## 3. Conclusions

We have discussed kilonova simulations, which have used a forward modelling approach to self-consistently simulate kilonovae from the merger through to synthetic observations. Through these simulations we have highlighted the importance of three-dimensional simulations when interpreting kilonova observations. A one-dimensional simulation with spherically averaged ejecta does not resemble the three-dimensional simulation in any viewing direction. Despite the total masses being the same in the one-dimensional and three-dimensional models, the bolometric light curve of the one-dimensional simulation is initially fainter than the three-dimensional simulation. The one-dimensional simulation is also much redder than the three-dimensional simulation. These differences could lead to uncertainties in inferring ejecta masses using one-dimensional models.

We have also shown the importance of accurate atomic data, both for simulating specific spectral features and for simulating the overall SED. We have demonstrated that by replacing only the atomic data for Sr, Y and Zr with experimentally calibrated data, the resulting spectra change dramatically. Moreover, using the calibrated data for Sr, Y and Zr improves agreement of the synthetic spectra with the observed spectra of AT2017gfo. Accurate and complete atomic line lists will be crucial for identifying kilonova spectral features and for predicting the overall colour evolution.

In the polar directions, our kilonova simulation showed a similar evolution to the observed spectra of AT2017gfo; however, the synthetic spectra evolved faster than the observations. Nonetheless, this simulation gives a prediction that kilonova spectra viewed edge-on near the equator will appear different from those observed for AT2017gfo and may be relatively featureless. We noted that the spectra near the equator are not significantly redder in colour temperature than those at the pole, despite the presence of lanthanide-rich material near the equator, suggesting kilonovae viewed edge-on could also show an early blue colour. However, this study was carried out for only a single merger ejecta model, considering one theoretical prescription for the equation of state and one binary neutron star mass ratio configuration. To understand the expected variation in kilonovae, this study needs to be expanded to investigate more models.

## Data Availability

The simulation code and analysis tools are available at [49].
